# Minneapolis Wins

**Published:** 1902-02

**Authors:** 


### MINNEAPOLIS WINS.

THE vote of the Executive Committee of the American Veterinary Medical Association has decided the place of meeting for 1902 in favor of Minneapolis, which will be the Association's first trip to the Northwest. Chicago and Detroit have been the nearest points to that city in the meetings of the past, and there will be much interest in the success of this meeting.

In selecting Minneapolis it is a recognition of one of the most substantial and successful cities of that wonderful section of our country. It is also an acknowledgment of the strength that Minnesota has developed in the profession, in greater numbers, in splendid association work, in broad sanitary control work, in educational legislation, in regulations by statute for the control and eradication of certain diseases so disastrous to the welfare of the live-stock industry, as well as the health of her people.

Her representatives of the profession have long been active in the American Veterinary Medical Association, and one could always depend upon that State being represented. They have always taken a keen interest in our work and contributed much to the success of our national organization. Since we have become American in name we must strive to become more wholly American in membership, and the Canadas and Manitoba will send more to our meeting in Minneapolis than probably to any other city in our States. It will be a successful meeting, and every member and veterinarian should plan to be present at Minneapolis in September. The names of Lyford, Reynolds, Brimhall, and McKenzie are guarantees of a good and profitable meeting.

